# Epidermal growth factor receptor (EGFR) mutations in non-small cell lung cancer (NSCLC) of Yunnan in southwestern China

**DOI:** 10.18632/oncotarget.14706

**Published:** 2017-01-17

**Authors:** Yongchun Zhou, Yanlong Yang, Chenggang Yang, Yunlan Chen, Changshao Yang, Yaxi Du, Guangqiang Zhao, Lianhua Ye, Yunchao Huang

**Affiliations:** ^1^ Tumor Research Institute of Yunnan Province, The Third Affiliated Hospital of Kunming Medical University (Yunnan Tumor Hospital), Kunming, 650118, PR China; ^2^ Department of Thoracic Surgery I, The Third Affiliated Hospital of Kunming Medical University (Yunnan Tumor Hospital), Kunming, 650118, PR China; ^3^ Department of Pathology, The Third Affiliated Hospital of Kunming Medical University (Yunnan Tumor Hospital), Kunming, 650118, PR China; ^4^ Cadre Ward, The Third Affiliated Hospital of Kunming Medical University (Yunnan Tumor Hospital), Kunming, 650118, PR China; ^5^ Key Laboratory of Lung Cancer Research of Yunnan Province, The Third Affiliated Hospital of Kunming Medical University, Kunming, 650118, PR China

**Keywords:** non-small cell lung cancer, EGFR mutation, cftDNA, Yunnan, Xuanwei

## Abstract

To investigate the Epidermal Growth Factor Receptor (EGFR) mutation status in non-small cell lung cancer (NSCLC) in Yunnan province in southwestern China, we detected EGFR mutation by Amplification Refractory Mutation System (ARMS) polymerase chain reaction (PCR) using DNA samples from 447 pathologically confirmed NSCLC specimens (175 tissue, 256 plasma and 16 cytologic samples). The relationship between EGFR mutations and demographic and clinical factors were further explored. Subgroup analyses according to sample type (tissue and plasma) and histological type (adenocarcinoma) were done. We found the mutation rate was 34.9% in overall patients (42.3%, 29.7%, and 37.5% for tissue, plasma, and cytologic samples respectively). We found female (*p* < 0.0001), no smoking (*p* = 0.001), adenocarcinoma (*p* < 0.0001), and tissue specimen (*p* = 0.026) were associated with higher EGFR mutation rate. The most common mutations were exon 19 deletions (40%) and L858R point (30%) mutation. Interestingly, NSCLC patients from Xuanwei harbored a strikingly divergent mutational pattern for EGFR when compared with non-Xuanwei patients (higher G719X, G719X+S768I mutations, but lower 19 deletion and L858R mutations). Generally, EGFR mutation rate and pattern in Yunnan province was in accord with other Asian populations. However, Xuanwei subgroup showed strikingly divergent EGFR mutation spectrum from other general population. Our analysis also indicated that cftDNA analysis for EGFR mutations detection was feasibility for the patients lacking sufficient tissue for molecular analyses.

## INTRODUCTION

Lung cancer is still the most common malignancy and is a leading cause of mortality worldwide. In China, Lung cancer had been becoming the most frequently diagnosed cancer (326,600 new cases with an incidence of 50.86/100,000) and the first leading cause of cancer death (with estimated deaths of 569,400) in 2012 [1]. Non-small cell lung cancer accounts for 85% of all lung cancer [2].

Platinum-based double-agent chemotherapy is the first-line therapy for patients with NSCLC [3]. However, for patients harboring active epidermal growth factor receptor (EGFR) mutations, EGFR tyrosine kinase inhibitors (TKIs) therapy may achieve better objective remission rate (ORR) and longer progression free survival(PFS) [4]. NCCN guideline suggested EGFR testing is strongly recommended in NSCLC, EGFR-TKIs are also recommended for NSCLC patients harboring sensitizing EGFR mutation as the first-line treatment [5]. Currently, tumor tissue, which is usually obtained by biopsy or surgery, is the gold standard for detection of EGFR mutations. Unfortunately, most NSCLC patients (70%) are diagnosed at an advanced stage and had no chance to receive surgery. Also, for patients with recurrence disease or acquired resistance to TKIs, repetition of a biopsy is not feasible and will increased discomfort for the patients. Thus, it is difficult to obtain sufficient tumor samples, and circulating-free tumor DNA (cftDNA) have emerged as an noninvasive and replicable method that could provide the same genetic information as a tissue biopsy. It could also be performed at any time during the course of therapy allowing for dynamic monitoring of molecular change [6].

Yunnan province, a region located in the Yunnan-Guizhou Plateau in southwestern China, with an average altitude of 2 kilometers. The natural geographical environment in Yunnan province is complicated and the subtropical mountainous and plateau areas accounts for 94% of Yunnan's total land territory (390,000km^2^). The total population of Yunnan includes 46 million people and consists of consisted of multi-ethnic groups including Yi (11%), Hani (3.5%), Bai (3.4%), Dai (2.6%), Zhuang (2.6%) and others (national census in 2011). Xuanwei City located in late Permian coal-accumulating areas in the eastern regions of Yunnan province. The incidence and mortality rate of lung cancer is the highest in China [7, 8]. The noticeable features of lung cancer in Xuanwei were: (1) the incidence and mortality rate of lung cancer in female was rather high and almost all of them did not smoke; (2) the major type of lung cancer in women was adenocarcinoma [9]. Previous studies indicated Xuanwei is located in late Permian coal-accumulating areas where is rich for bituminous (smoky) coal. The main cause of high incidence and mortality of lung cancer is indoor air pollution caused by the use of “smoky coal”, which releases carcinogenic substances such as polycyclic aromatic hydrocarbons (PAHs), particulate matter and crystalline quartz [7, 10, 11]. As unique environment, ethnic group and certain susceptible population may have certain genetic background. Investigating EGFR mutation profile of NSCLC patients in Yunnan province especially in Xuanwei region is meaningful.

## RESULTS

### Baseline characters of included patients

Four hundred and forty-seven pathologically confirmed NSCLC patients were included in our analysis. Among these patients, 201 (45.0%) were females and 246 (55.0%) were males. The mean age was 58.2 ± 11.33 years, ranging from 27 to 86 years. Two hundred and twelve patients (47.4%) were smokers and the remaining 235 were never-smokers. 35 (7.8%) patients had the family history of malignancy. The most common histology was adenocarcinoma (387, 86.6%), and the remaining were squamous carcinoma (39, 8.7%), adenosquamous carcinoma (6, 1.3%), large cell carcinoma (4, 0.89%), and NSCLC type undetermined in 11 (2.5%) patients. 8 (1.8%) patients had a history of EGFR-TKI treatments. According to 8 regions of Yunnan, 135 were from central region (Kunming, Yuxi), 118 were from east region (Qujing), 31 were from northeast region (Zhaotong), 35 were from southeast (Wenshan), 15 were from south region (Honghe), 48 were from west region (Chuxiong, Dali, Nujiang, Baoshan, Dehong), 41 were from northwest region (Lijiang, Shangri-La), and the remaining 34 were from southwest region (Xishuangbanna, Lincang, Puer). 63 patients were from Xuanwei where the incidence and mortality rate of lung cancer were the highest in China. Han accounted for the most common ethnic group (406, 90.8%), and the remaining were Yi (14, 3.1%), Bai (7, 1.6%), Dai (5, 1.1%), Naxi (3, 0.67%), Hui (4, 0.89%), Buliang (2, 0.45%), Lisu (2, 0.45%) and Tujia (1, 0.22%). 175 patients with tissue (23 were obtain from biopsy, and 152 were obtain from surgical specimen), 16 patients with cytology specimen (pleural fluid) were available for EGFR mutation analysis. For the remaining 256 patient, most of them were stage IV (222, 86.7%) and could not provide sufficient tissue samples for EGFR testing, plasma were obtained for EGFR testing. Table 1 summarized the main baseline characters of included patients.

**Table 1:** Frequency of EGFR mutation according to clinical characteristics in overall patients

	Positive			Negative		*P*
	*N*	*N*	%	*N*	%	
**Age**						0.272
< 65	310	112	36.1	198	64	
65–75	105	37	35.2	68	65	
> 75	32	7	21.9	25	78	
**Sex**						< 0.0001
Male	246	63	25.6	183	74	
Female	201	93	46.3	108	54	
Smoking						0.001
Yes	212	58	27.4	154	73	
No	235	98	41.7	137	58	
**Family history of malignancy**						0.452
Yes	35	13	37.1	22	63	
No	412	143	34.7	269	65	
**Histology**						< 0.0001
AD	387	149	38.5	238	62	
SCC	39	5	12.8	34	87	
ADSC	6	1	16.7	5	83	
LCC	4	0	0	4	100	
NSCLC	11	1	9.1	10	91	
**Tumor Site**						0.137
Left	203	61	30	142	70	
Right	242	94	38.8	148	61	
Bilateral	2	1	50	1	50	
**Region distribution**						0.209
Central	135	35	25.9	100	74	
East	118	51	43.2	67	57	
Northeast	31	12	38.7	19	61	
Southeast	35	11	31.4	24	69	
South	15	6	40	9	60	
West	38	13	34.2	25	66	
Northwest	41	14	34.1	27	66	
Southwest	34	14	41.2	20	59	
**Ethnic**						0.19
Han	406	145	35.7	261	64	
Yi	14	5	35.7	9	64	
Bai	7	3	42.9	4	57	
Dai	5	1	20	4	80	
Naxi	3	0	0	3	100	
Hui	4	0	0	4	100	
Buliang	2	0	2	2	100	
Lisu	2	0	2	2	100	
Tujia	1	0	1	1	100	
**Stage**						0.278
Ia	20	10	50	10	50	
Ib	30	13	43.3	17	57	
IIa	10	3	30	7	70	
IIb	12	7	58.3	5	42	
IIIa	38	11	28.9	27	71	
IIIb	34	9	26.5	25	74	
IV	303	103	34	200	66	
**Brain metastasis**						0.203
Yes	64	27	42.2	37	58	
No	383	129	33.7	254	66	
**Sample type**						0.026
Tisue	175	74	42.3	101	58	
Plasma	256	76	29.7	180	70	
Cytology	16	6	37.5	10	63	
**Xuanwei origin**						0.157
Yes	63	27	42.8	36	57	
No	384	129	33.6	255	66	
**Total**	447	156	34.9	291	65	

Note: AD, adenocarcinoma; SCC, squamous-cell carcinoma; ADSC, adenosquamous carcinoma; LCC, large cell carcinoma; NSCLC, non-small-cell lung cancer.

### EGFR mutation rates in tissue and plasma for patients who provided both samples

To explore the feasible and consistency of EGFR mutation detection in cftDNA in our center, 29 patients provided both tissue and plasma were used to analysis the consistency of EGFR mutation detection in cftDNA when compared with tissue. EGFR mutations were detected in 8 (27.6%) tumor tissue samples, of which, three harbored 19del, two harbored L858R and one harbored 20ins. The EGFR mutation status of matched tissue and plasma were concordant for 25 patients (positive *n* = 5, negative *n* = 20, κ coefficient 0.626, *p* = 0.001). The sensitivity of plasma was 67.5% (5/8), the specificity was 95.2% (20/21), the positive predictive value (PPV) was 83% (5/6), and the negative predictive value (NPV) was 87.0 (20/23). Our data suggest that detection of EGFR mutations in cftDNA is relatively sensitive and highly specific in our center.

### Incidence of EGFR mutation and its association with demographic and clinical factors

EGFR mutation frequency and its relationship with clinicopathological parameters in NSCLC patients in Yunnan are similar to other East Asian countries.

The EGFR mutation was detected in 156 NSCLC patients (34.9%). The difference in EGFR mutation rate was found according to sex, smoking status, pathology type and sample type. It seemed that female (*p* < 0.0001), no smoking (*p* = 0.001), adenocarcinoma (*p* < 0.0001), and tissue specimen (*p* = 0.026) were associated with higher EGFR mutation. However, no significant association was found in age (*p* = 0.272), family history of malignancy (*p* = 0.452), the site of tumor (0.137), TNM stage (*p* = 0.278), brain metastasis (*p* = 0.203), the distribution of region (*p* = 0.209), ethnic (*p* = 0.190) and Xuanwei origin (*p* = 0.157).

### Incidence of EGFR mutation in tissue, plasma and adenocarcinoma subgroups

Subgroup analysis suggested although the overall mutation rate is different in tissue, plasma and adenocarcinoma subgroups, the relationship between EGFR mutations and clinicopathological parameters was similar.

As sample type maybe a confounding factor for EGFR detecting, we investigated the Incidence of EGFR mutation in tissue and plasma groups respectively. The EGFR mutation rate was 42.3% (74/175), 29.7% (76/256) in tissue and plasma respectively. In tissue subgroups, younger age (< 65 year) (*p* < 0.001), female (*p* < 0.001), no smoking status (*p* < 0.001), and adenocarcinoma (*p* < 0.001) was correlated with higher EGFR mutation rate. Also, distribution of region (*p* = 0.007), Xuanwei origin (*p* = 0.007) also had an impact in EGFR mutation rate. In plasma subgroup, we found female (*p* < 0.001), no smoking (*p* = 0.01) and adenocarcinoma (*p* = 0.042) was associated with higher EGFR mutation rates (Table 2).

**Table 2:** Frequency of EGFR mutation in tissue and plasma subgroups

		Tissue					Plasma					
	Positive		Negative				Positive		Negative		
N	N	%	N	%	P	N	N	%	N	%	P
Age						< 0.0001						0.27
< 65	122	58	47.5	64	52.5		177	50	28	127	72	
65–75	44	16	36.4	28	63.6		57	20	35	37	65	
> 75	9	0	0	9	100		22	6	27	16	73	
Sex						< 0.0001						< 0.0001
Male	93	26	28	67	72		142	33	23	109	77	
Female	82	48	58.5	34	41.5		114	43	38	71	62	
Smoking						0						0
Yes	82	24	29.3	58	70.7		121	31	26	90	74	
No	93	50	53.8	43	46.8		135	45	33	90	67	
Family history						0.85						0.45
Yes	15	6	40	9	57.1		19	7	37	12	63	
No	160	68	42.5	92	57.5		237	69	29	168	71	
Histology						< 0.0001						0.04
AD	147	71	48.3	76	51.7		331	73	32	158	68	
non-AD	28	3	10.7	25	89.3		25	3	12	22	88	
Tumor site						0.44						0.14
Left	84	33	39.3	51	60.7		112	26	23	86	77	
Right	91	41	45.1	50	54.9		143	49	34	94	66	
Bilateral							1	1	100	0	0	
Region distribution						0.01						0.96
Central	56	14	25	42	75		71	18	25	53	75	
East	47	29	61.7	18	38.3		69	20	29	49	71	
Northeast	12	7	58.3	5	41.7		19	5	26	14	74	
Southeast	14	4	28.6	10	71.4		20	6	30	14	70	
South	5	3	60	2	40		10	3	30	7	70	
West	14	5	35.7	9	64.3		22	8	36	14	64	
Northwest	17	6	35.3	11	64.7		23	8	35	15	65	
Southwest	10	6	60	4	40		22	8	36	14	64	
Ethnic						0.31						0.73
Han	160	70	43.8	90	56.3		231	70	30	161	70	
non-Han	15	4	26.7	11	73.3		25	6	24	19	76	
Stage						0.79						0.44
I-IIIa	99	45	50	57	50		10	2	20	8	80	
IIIb-IV	76	32	42.4	44	57.6		246	74	31	172	69	
Brain metastasis						0.14						0.2
Yes	21	12	57.1	9	42.9		43	15	35	28	65	
No	154	62	40.3	92	59.7		213	61	29	152	71	
Xuanwei origin						0						0.16
Yes	28	19	67.8	9	32.2		35	8	23	27	77	
No	147	55	37.4	92	62.6		221	68	31	153	69	
Total	175	74	42.3	101	57.7		256	76	30	180		70.3

We also analyzed the frequency of EGFR mutation in adenocarcinoma subgroup. The incidence of EGFR mutation was 38.5% (149/387) in adenocarcinoma. Female (*p* = 0.001), no smoking status (*p* = 0.016), and tissue specimen (*p* = 0.006) may associated with higher EGFR mutation rate (Table 3).

**Table 3:** Frequency of EGFR mutation in adenocarcinoma

		Positive		Negative		*P*
	*N*	*N*	%	*N*	%	
Age						0.61
< 65	276	107	38.3	169	61	
65–75	87	35	40.2	52	60	
> 75	24	7	29.2	17	71	
**Sex**						0.001
Male	200	61	30.5	139	70	
Female	187	88	47.1	99	53	
**Smoking**						0.016
Yes	170	54	31.8	116	68	
No	217	95	43.8	122	56	
**Family history of malignancy**						0.98
Yes	31	12	38.7	19	61	
No	356	137	38.5	219	62	
**Tumor Site**						0.284
Left	170	58	34.1	112	66	
Right	215	90	41.9	125	58	
Bilateral	2	1	50	1	50	
**Region distribution**						0.095
Central	110	33	30	77	70	
East	110	50	45.5	60	55	
Northeast	28	11	39.3	17	61	
Southeast	28	11	39.3	17	61	
South	14	6	42.9	8	57	
West	32	12	37.5	20	63	
Northwest	36	13	36.1	23	64	
Southwest	29	13	44.8	16	55	
**Ethnic**						0.131
Han	353	140	39.7	213	60	
non-Han	34	9	26.5	25	74	
**Stage**						0.124
I-IIIa	88	42	55.6	46	44	
IIIb-IV	299	107	36.8	192	63	
**Brain metastasis**						0.314
Yes	61	27	44.3	34	56	
No	326	122	37.4	204	63	
**Sample type**						0.006
Tissue	144	70	48.6	74	51	
Plasma	228	73	32	155	68	
Cytology	15	6	40	9	60	
**Xuanwei origin**						0.26
Yes	60	27	45	33	55	
No	327	122	37.3	205	63	
**Total**	387	149	38.5	238	61.5	

### Types of EGFR mutation

EGFR mutation pattern in Yunnan province was in accord with other Asian populations. In Xuanwei subgroup, we found the prevalence of EGFR mutation was different from other general population (higher G719X, G719X+S768I, but lower 19 deletion and L858R mutations).

Overall, EGFR mutation was detected in 156 patients. The most common mutations were exon 19 deletion and L858R point mutation, which was observed in 63 (40%) and 46 (30%) patients, respectively. Single mutation was observed in 139 patients (89.1%), and combined mutation was found in 17 patients (11.9%). Among 156 patients, 127 patients (81.4%) harbored sensitizing mutations, 17 patients (10.9%) harbored resistant mutations, and the remaining 12 patients had both sensitizing and resistant mutations. Eight patients harbored single T790M mutation its combined mutations (three patients for T790M, two for S768I+T790M, and three for 19-del+T790M respectively.). Among these patients, four had ever received EGFR-TKI therapy. Other four patients had ever received TKI treatment were wildtype. Except them, the reaming 339 patients had never received TKI treatment before.

**Figure 1:**
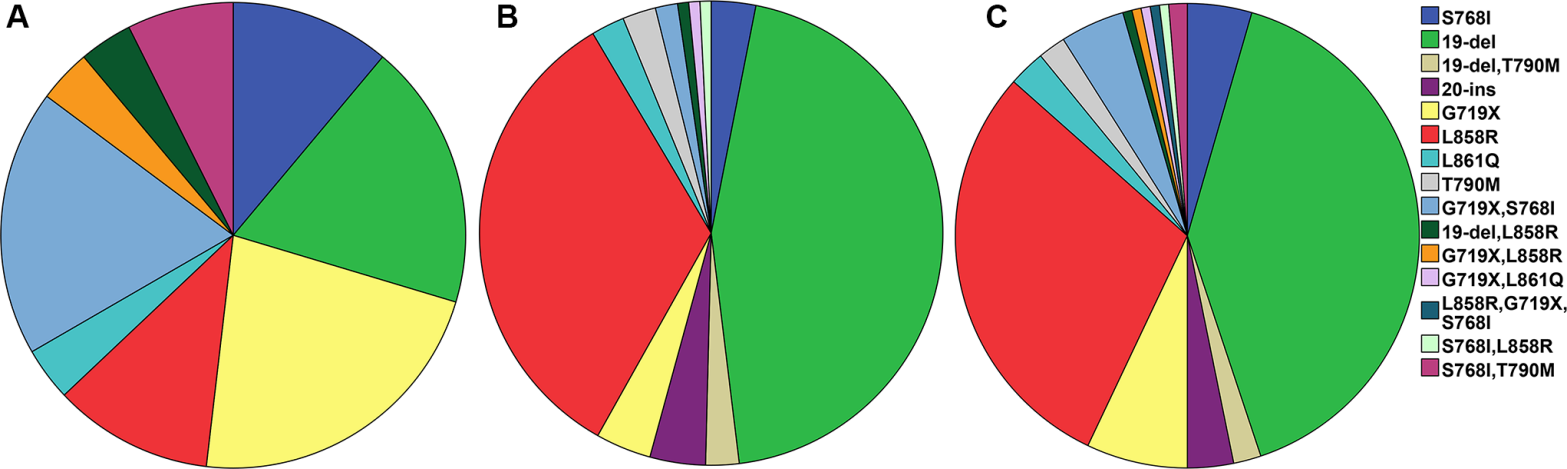
The EGFR mutation spectrum in (**A**) Xuanwei population, (**B**) non-Xuanwei population, (**C**) overall population NSCLC patients in Xuanwei had higher G719X, G719X+S768I, but lower 19 deletion and L858R mutations when compared with non-Xuanwei patients.

We also performed the subgroup analysis to explore whether sample type, Xuanwei origin, sex, and smoking status would affect the distribution of EGFR mutation type. Our analysis indicated that, the EGFR mutation type distribution in Xuanwei origin was different in other population in Yunnan province. It seemed the prevalence of G719X, S768I+T790M, and G719X+S768I mutations were more common in Xuanwei origin than in other population in Yunnan provinces. However, NSCLC patients with Xuanwei origin harbored lower 19-deletion and L858R mutation rate when compared with other population in Yunnan province (Table 4 and Figure 1). According to sample type, we found the frequency of 19-deletion in tissue sample was higher than in plasma. No difference was found in the distribution of EGFR mutation type according to sex and smoking status (Table 4).

**Table 4:** EGFR mutation type in our analysis

			Xuanwei					Sample type					Sex					Smoking				
		Yes		No			Tissue		Plasma			Male		Female			Yes		No		
N	%	N	%	N	%	P	N	%	N	%	P	N	%	N	%	P	N	%	N	%	P
Sensitizing mutations																						
G719X	11	7.05	6	22.2	5	3.9	0.001	8	11	3	3.8	0.12	6	9.4	5	5.4	0.34	6	9.8	5	5.3	0.276
19-deletion	63	40.4	5	18.5	58	45	0.019	23	31.5	38	47.5	0.04	28	35	35	38	0.58	22	36.1	41	43.2	0.378
L861Q	4	2.6	1	3.7	3	2.3	0.54	3	4.1	1	1.3	0.35	3	4.7	1	1.1	0.306	3	4.9	1	1.1	0.3
L858R	46	29.5	3	11.1	43	33.3	0.038	23	31.5	23	28.8	0.71	16	25	30	32.6	0.305	19	31.1	27	28.4	0.813
G719X, L861Q	1	0.6	0	0	1	0.8	0.10	1	1.4	0	0	0.48	0	0	1	1.1	1.00	0	0	1	1.1	1.00
G719X, L858R	1	0.6	1	3.7	0	0	0.173	1	1.4	0	0	0.48	0	0	1	1.1	1.00	0	0	1	1.1	1.00
19-deletion, L858R	1	0.6	0	0	1	0.8	0.10	0	0	1	1.3	1.00	0	0	1	1.1	1.00	0	0	1	1.1	1.00
Resistance mutations																						
T790M	3	1.9	0	0	3	2.3	0.10	1	1.4	2	2.5	1.00	0	0	3	3.3	0.269	1	1.6	2	2.1	1.00
S768I	7	4.5	3	11.1	4	3.1	0.10	4	5.5	3	3.8	0.71	5	7.8	2	2.2	0.124	4	6.6	3	3.2	0.433
20-insertion	5	3.2	0	0	5	3.9	0.588	2	2.7	2	2.5	1	3	4.7	2	2.2	0.401	3	4.8	2	2.1	0.38
S768I,T790M	2	1.3	2	7.4	0	0	0.029	0	0	2	2.5	0.5	2	31	0	0	0.167	2	3.3	0	0	0.151
Combination of sensitizing and resistance mutations																						
19-del, T790M	3	1.9	0	0	3	2.3	1.00	0	0	3	100	0.25	0	0	3	3.3	0.269	0	0	3	3.2	0.281
G719X, S768I	7	4.5	5	18.4	2	1.6	< 0.0001	5	6.8	2	2.5	0.26	1	1.6	6	6.5	0.241	1	1.6	6	6.3	0.248
S768I, L858R	1	0.6	0	0	1	0.8	0.10	1	1.4	0	0	0.48	0	0	1	1.1	1.00	0	0	1	1.1	1.00
L858R, G719X, S768I	1	0.6	1	3.7	0	0	0.17	1	1.4	0	0	0.48	0	0	1	1.1	1.00	0	0	1	1.1	1.00

## DISCUSSION

In the present study, we investigated the prevalence of EGFR mutation rate in Yunnan province, a mountainous and plateau areas consisted of multi-ethnic groups.

### EGFR mutation rate and pattern in overall population

The EGFR mutation rate was 34.9% and 42.3% among patients with NSCLC and adenocarcinoma, respectively. In tissue sample, the mutation rate was much higher (42.3% for overall NSCLC patients, and 48.6% for adenocarcinoma). The frequency was in the range of other reports in East Asian countries (31%-50%) [12–16]. Also, female, never-smokers and adenocarcinoma was correlated with higher rate of NSCLC patients was observed in overall and various subgroups in our studies. This was also familiar with other previous studies [13–15].

In our study, the most frequent mutation patterns of EGFR were 19 deletion and L858R, which was similar to that reported in studies performed in East Asian countries [12–15]. In our study, the prevalence of T790M mutation was relatively higher than reported [the mutation rates of single T790M mutation and complex EGFR mutation containing T790M accounted for 1.9% (three patients) and 3.2% (eight patients) among total mutations]. We supposed two reasons may account for this issue. Firstly, half of these eight patients had ever received TKI treatment, as a result, the secondary T790M mutation may relate to acquired TKIs resistance in these four patients. Besides, our study recruited patients in single center and the sample size was not large enough, so our sample could not better reflect the overall population.

### EGFR mutation pattern in Xuanwei

Hosgood et al. reported that the incidence of G719X mutations in exon 18 was higher than general population (50% versus 4%), but L858R mutations was lower than general population (14% versus 41%) [17]. The study by Chen et al. suggested that when compared with patients from non-Xuanwei areas, the NSCLC patients from Xuanwei area harbored higher frequency of G719X+S768I in exon 18 and 21 (45.1% versus 4.1%, *p* < 0.0001), but had lower frequency of 19 deletion (7.8% versus 49.3%, *p* < 0.0001) [18]. In our study, we found when compared with non-Xuanwei population, NSCLC patients in Xuanwei areas had higher G719X, G719X+S768I mutations, but harbored lower 19 deletion and L858R mutations. Previous positive findings in Xuanwei patients could be repeated by our analysis. Additionally, Previous studies showed rare EGFR mutations (G719X or L861Q) may had shorter overall survival when compared with those harboring “classical” EGFR mutations (19 deletion or L858R) [19]. However, other rare mutations rather than G719X and L861Q would lead to a worse response to EGFR TKIs [20–22]. We supposed the prognosis of NSCLC patients in Xuanwei harboring EGFR mutation was theoretically not as better as other populations received EGFR-TKIs treatment.

The EGFR mutation spectrum in Xuanwei was different from other population and even different from never smoking female populations in China. Hosgood et al. supposed this difference might be caused by exposing to indoor air pollution from local smoky coal [17]. Burning “smoky coal” would releases high concentration of PAHs. In Xuanwei residents buring “smoky coal”, PAH-DNA adducts have also been observed in bronchoalveolar lavage [23]. Cell-line studies suggested PAHs would increase intracellular calcium in human cell lines, thus may lead to EGFR-dependant cell proliferation [24]. Similarly, KRAS and TP53 mutation spectra in nonsmokers in Xuanwei was consistent with an exposure to PAH, but different with those smokers [25]. Salmonella exposed by smoky coal emissions showed similar KRAS and TP53 mutation spectra exposed by PAHs [26]. These results show that mutations in the TP53 and KRAS genes can reflect a specific environmental exposure. As a result, the unique EGFR mutation spectrum in Xuanwei areas might be related to the exposure of air pollution from local smoky coal. However, which component may play the dominating role, PAHs, particulate matter, crystalline quartz, or their interactions? What is the potential mechanism? Further studies are expected.

### The feasibility of cftDNA analysis for EGFR mutations detection

EGFR mutation analysis is necessary for drug prescription purpose and therefore tumor tissue is always required. Unfortunately, biopsies (bronchoscopy and trans-thoracic biopsies) are not well accepted because tumor tissue is not sufficient or adequate for molecular analysis [27]. Also, repetition of a biopsy would bring patients discomfort. cftDNA has emerged as promising candidate for dynamic monitoring of molecular change [28]. To validate cftDNA analysis for EGFR mutations detection, some efforts have been made in comparing the feasibility of cftDNA analysis for EGFR mutations detection with the actual gold standard that is analysis on tissue. A meta-analysis by Qiu et al. included 27 studies involving 3,110 participants. They reported pooled sensitivity and specificity of cftDNA in detecting EGFR mutation were 0.620 (95% CI: 0.513–0.716) and 0.959 (95% CI: 0.929–0.977), respectively and area under the curve (AUC) was 0.91 (95% CI: 0.89–0.94) [29]. In ASSESS trial, 1,311 patients were enrolled with data available on both tissue and plasma samples of 1,162, the concordance was 89.1%, with a sensitivity of 46%, specificity of 97.4%, PPV of 77.7% and NPV of 90.3% [30]. All this evidence is in favor of the high diagnostic accuracy of cftDNA underlying the high specificity and non-invasivity that make it a useful tool for screening. In our study, 29 patients with paired tissue and plasma were available for analysis. The sensitivity was 67.5%, the specificity was 95.2%, the PPV was 83%, and the NPV was 87.0%. However, smaller sample size may lead to bias. Anyway, we successfully detected EGFR mutation in cftDNA in 256 patients. Most of these patients were stage IV (86.7%) and tumor tissues are not sufficient for EGFR mutation analysis. Disease stage was significantly associated with detecting sensitivity. For patients with advanced stage, the sensitivity of EGFR mutation detection by cftDNA was rather higher than in early stage [31, 32]. So, we applied EGFR detection in cftDNA in advanced stage was scientifically reasonable.

To avoid bias, we also analyzed the EGFR mutation rate and pattern in tissue and plasma subgroup. And we found except the overall mutation rate in plasma was lower, the distribution pattern of EGFR mutations was similar to tissue group and previous reports. Our center confirmed the feasible of EGFR mutation detection in cftDNA. It would bring a group of patients a huge benefit from targeted mutation identification for whom obtaining tissue sample is sometimes not feasible. Gives the chance of a targeted therapy also in patients who cannot undergo invasive diagnostic procedures, due to comorbidities or the absence of biopsable tumor lesions

### Limitations

Some issues should be acknowledged.

Firstly, our data could not represent the true prevalence of EGFR mutation in the NSCLC patients in Yunnan provinces. We only collected available data in our center which may lead to a selection bias. In our study, we found most of our patients were from central and east region (56.6%) of Yunnan. Although Yunnan was a multi-ethnic region, Han accounted for 90.8% of our included patients. The sample from other ethnic patient was lacking, which could not reflect the actually genetic background of Yunnan residents. Also, the sample size of our study was not large enough to reflect the overall population.

Besides, cftDNA analysis for EGFR mutations detection could not reflect actually prevalence of EGFR mutation. Detecting EGFR mutation in tumor tissue was gold standard. As we show above, although the specificity of ctfDNA was rather higher, the sensitivity of ctfDNA was 0.620 (95% CI: 0.513–0.716), and even lower in two trial. As a result, the EGFR mutation rate in plasma was rather lower than in tissue. Some patients with truly EGFR mutation may not detected by ctfDNA. In our study, most of sample analyzed were obtained from plasma (57.3%). And the mutation rate was lower than in tissue (29.7% versus 42.3%, *p* = 0.026). This may affected the true prevalence of EGFR mutation. Detecting EGFR mutation in cftDNA cannot totally substitute for a tumor biopsy. The positive results of EGFR mutation status detected in plasma are highly reliable. Due to the high false negative rate in blood samples, the negative results of EGFR mutation status in plasma need further confirmation.

At last, we did not examine EGFR gene amplification or protein of wild-type and activated status in our study. EGFR mutations, gene amplification, and protein expression may not correlate with each other [33]. EGFR mutation is a better predictive marker for TKIs therapy compared to EGFR gene amplification and protein expression [33, 34]. Activating mutations of the EGFR may increase the receptor activity even in the absence of protein overexpression [33]. EGFR mutations change the configuration of the kinase to affect the efficacy of TKIs [35]. Detecting EGFR amplification and protein in matching wild-type and activated status may contribute towards better mechanics exploration for lung cancer development in Yunnan.

## MATERIALS AND METHODS

### Study population

Patients with pathologically confirmed NSCLC who visited the Third Affiliated Hospital of Kunming Medical University between August 2015 and July 2016 were enrolled in our study. Eligibility criteria were:1) adults(> 18 year) who were residents of Yunnan province, 2) histologically or cytologically confirmed NSCLC. Written informed consents were obtained from all included individuals and approval for this study was obtained from the ethical committee of the Third Affiliated Hospital of Kunming Medical University.

### DNA extraction

Tissue samples were obtained from excision specimens and biopsy specimens (bronchoscopic biopsy, transbronchial lung biopsy, percutaneous needle biopsy, pleural biopsy and biopsy of metastatic sites). Cytology was mainly obtained from pleural fluids. DNA was extracted from tissue and cytology using an AmoyDx tissue/pleural fulid DNA Kit (Amoy Diagnostics, Xiamen, China) according to the manufacturer's instructions. Plasma was separated from 10 ml of peripheral blood in EDTA anticoagulant tubes by centrifugation at 3000 rpm for 5 min within 2 h after collection and stored at −80°C until DNA extraction. Plasma DNA was isolated using an AmoyDx Circulating DNA Kit.

### Mutational analysis

Extracted DNA from the tissue and plasma samples were used for the detection of EGFR mutation by Human EGFR Gene Mutation Fluorescence Polymerase Chain Reaction (PCR) Diagnostic Kit (Amoy Diagnostics, Xiamen, China). 29 known mutations in EGFR exons 18–21 were analyzed (Supplementary Table S1). The Kit was based on amplification refractory mutation system (ARMS) technology. AmoyDx EGFR Mutations Detection Kit (Amoy Diagnostics, Xiamen, China) had been approved for clinical usage by China Food and Drug Administration (CFDA) since 2010.

### Statistical analysis

The relationship between EGFR mutation and demographic and clinical factors (such as age, sex, smoking status, histological type, population distribution, ethnic, specimen type, tumor site and whether Xuanwei origin or not, et.al) were analyzed by Pearson Chi-square or Fisher exact test were. All the statistics were performed by SPSS 22.0 (SPSS Inc., Chicago, IL, USA), two-sided *p* < 0.05 were considered statistically significant.

## CONCLUSIONS

Overall, the prevalence of EGFR mutation in NSCLC patients in Yunnan province was consistent with other Asian populations. In Xuanwei subgroup, we found the prevalence of EGFR mutation was different from other general population (higher G719X, G719X+S768I, but lower 19 deletion and L858R mutations). Besides, EGFR mutation analysis by cftDNA is feasible and represents one of the most important recent breakthroughs in thoracic oncology. With the limitation of our study, large sample size from multi-center in Yunnan should be included to make it more representative of the overall population. Also, as tumor tissue is gold standard to detect EGFR mutation, it should be collected as possible.

### Supplementary data

Supplementary Table 1. 29 known mutations in EGFR exons 18–21 analyzed in our study.

## SUPPLEMENTARY MATERIALS FIGURES AND TABLES


