# Supplementary material for: Epidermal growth factor receptor (EGFR) mutations in non-small cell lung cancer (NSCLC) of Yunnan in southwestern China
Source: Oncotarget. 2017 Jan 17;8(9):15023–33. doi: 10.18632/oncotarget.14706 (PMC5362464; doi:10.18632/oncotarget.14706)
Supplement: Supplementary file 1 [file oncotarget-08-15023-s001.pdf]

Epidermal growth factor receptor (EGFR) mutations in non-small cell lung cancer (NSCLC) of Yunnan in southwestern china

Supplementary Materials

Supplementary Table S1: 29 known mutations in EGFR exons 18–21 analyzed in our study

Mutation type	Mutation site	Exon	Changing in base
Ex18-mutant-1	G719A	18	2156G > C
Ex18-mutant-2	G719S	18	2155G > A
Ex18-mutant-3	G719C	18	2155G > T
Ex19-mutant-1	E746_A750del (1)	19	2235_2249del15
Ex19-mutant-2	E746_A750del (2)	19	2236_2250del15
Ex19-mutant-3	L747_P753 > S	19	2240_2257del18
Ex19-mutant-4	E746_T751 > I	19	2235_2252 > AAT(complex)
Ex19-mutant-5	E746_T751del	19	2236_2253del18
Ex19-mutant-6	E746_T751 > A	19	2237_2251del15
Ex19-mutant-7	E746_S752 > A	19	2237_2254del18
Ex19-mutant-8	E746_S752 > V	19	2237_2255 > T(complex)
Ex19-mutant-9	E746_S752 > D	19	2238_2255del18
Ex19-mutant-10	L747_A750 > P	19	2238_2248 > GC(complex)
Ex19-mutant-11	L747_T751 > Q	19	2238_2252 > GCA(complex)
Ex19-mutant-12	L747_E749del	19	2239_2247del9
Ex19-mutant-13	L747_T751del	19	2239_2253del15
Ex19-mutant-14	L747_S752del	19	2239_2256del18
Ex19-mutant-15	L747_A750 > P	19	2239_2248TTAAGAGAAG > C(complex)
Ex19-mutant-16	L747_P753 > Q	19	2239_2258 > CA(complex)
Ex19-mutant-17	L747_T751 > S	19	2240_2251del12
Ex19-mutant-18	L747_T751del	19	2240_2254del15
Ex19-mutant-19	L747_T751 > P	19	2239_2251 > C(complex)
Ex20-mutant-1	T790M	20	2369C > T
Ex20-mutant-2	S768I	20	2303G > T
Ex20-mutant-3	H773_V774insH	20	2319_2320insCAC
Ex20-mutant-4	D770_N771insG	20	2310_2311insGGT
Ex20-mutant-5	V769_D770insASV	20	2307_2308insgccagcgtg
Ex21-mutant-1	L858R	21	2573T > G
Ex21-mutant-2	L861Q	21	2582T > A
